# High Fructose Intake and Adipogenesis

**DOI:** 10.3390/ijms20112787

**Published:** 2019-06-07

**Authors:** Adrián Hernández-Díazcouder, Rodrigo Romero-Nava, Roxana Carbó, L. Gabriela Sánchez-Lozada, Fausto Sánchez-Muñoz

**Affiliations:** 1 Departamento de Inmunología, Instituto Nacional de Cardiología Ignacio Chávez, Mexico city 14080, Mexico; adrian.hernandez.diazc@hotmail.com; 2 Departamento de Ciencias de la Salud, Área de Investigación Médica, Universidad Autónoma Metropolitana Iztapalapa, Mexico city 09340, Mexico; roloromer@gmail.com; 3 Laboratorio de investigación en Farmacología, Hospital Infantil de México Federico Gómez, Mexico city 06720, Mexico; 4 Sección de Postgraduados, Escuela Superior de Medicina, Instituto Politécnico Nacional, Mexico city 11340, Mexico; 5 Departamento de Biomedicina Cardiovascular, Instituto Nacional de Cardiología Ignacio Chávez, Mexico city 14080, Mexico; roxcarbo@gmail.com; 6 Laboratorio de Fisiopatología Renal, Departamento de Nefrología, Instituto Nacional de Cardiología Ignacio Chávez, Mexico city 14080, Mexico; lgsanchezlozada@gmail.com

**Keywords:** fructose, adipogenesis, glucocorticoids, ROS, microRNAs, adipose tissue, angiotensin II, uric acid

## Abstract

In modern societies, high fructose intake from sugar-sweetened beverages has contributed to obesity development. In the diet, sucrose and high fructose corn syrup are the main sources of fructose and can be metabolized in the intestine and transported into the systemic circulation. The liver can metabolize around 70% of fructose intake, while the remaining is metabolized by other tissues. Several tissues including adipose tissue express the main fructose transporter GLUT5. In vivo, chronic fructose intake promotes white adipose tissue accumulation through activating adipogenesis. In vitro experiments have also demonstrated that fructose alone induces adipogenesis by several mechanisms, including (1) triglycerides and very-low-density lipoprotein (VLDL) production by fructose metabolism, (2) the stimulation of glucocorticoid activation by increasing 11β-HSD1 activity, and (3) the promotion of reactive oxygen species (ROS) production through uric acid, NOX and XOR expression, mTORC1 signaling and Ang II induction. Moreover, it has been observed that fructose induces adipogenesis through increased ACE2 expression, which promotes high Ang-(1-7) levels, and through the inhibition of the thermogenic program by regulating Sirt1 and UCP1. Finally, microRNAs may also be involved in regulating adipogenesis in high fructose intake conditions. In this paper, we propose further directions for research in fructose participation in adipogenesis.

## 1. Introduction

Modern societies, especially those with a high incidence of obesity, are characterized by high fructose intake [1]. Most of this high fructose intake comes from the consumption of added sugar products [2,3]. Both sucrose (glucose-fructose disaccharide), or high fructose corn syrup (HFCS, free fructose and glucose at different concentrations) are primary sources of fructose in the diet.

There is a vast body of evidence of the harmful effects of excessive intake of fructose in the liver and other tissues [4,5,6]. However, the role of fructose in adipogenesis is less known. Therefore, the purpose of this review is to give some insight into the effects of fructose on white adipose tissue (WAT) cells, specifically on the adipogenic programs. In this regard, some studies have reported WAT expansion in response to high fructose intake [7,8]. In overweight and obese subjects, the consumption of 25% of daily energy requirement as fructose, provided as a sweetened beverage, for 10 weeks increased the visceral adiposity as compared to those who consumed an isocaloric glucose-sweetened beverage [8]. Supporting this notion, a 6-year prospective study in the Framingham’s Third Generation Cohort showed that those individuals who drink sugar-sweetened beverages (SSBs) on a daily basis had 29% more visceral WAT than the non-consumers [9]. Thus, high fructose consumption in the form of SSBs contributes to WAT expansion. Studies have long shown that fructose acute absorption can occur in white adipose tissue [10,11]. There is also evidence that fructose on its own induces the differentiation of adipocytes in vitro [12,13]. This has been shown in cell lines as well as in primary cultures [14,15].

The increased exposure of WAT and the cellular metabolic adaptations to chronic fructose intake target several mechanisms involved in the formation of new adipocyte cells. In the present review, we revisit how fructose induces glucocorticoids (GCs), activates the production of reactive oxygen species (ROS), and activates the renin–angiotensin system (RAS) and the induction of microRNAs (miRNAs) as an adipogenic mechanism. Finally, further directions in research are proposed.

## 2. Fructose Absorption to the Systemic Circulation

**Figure 1:**
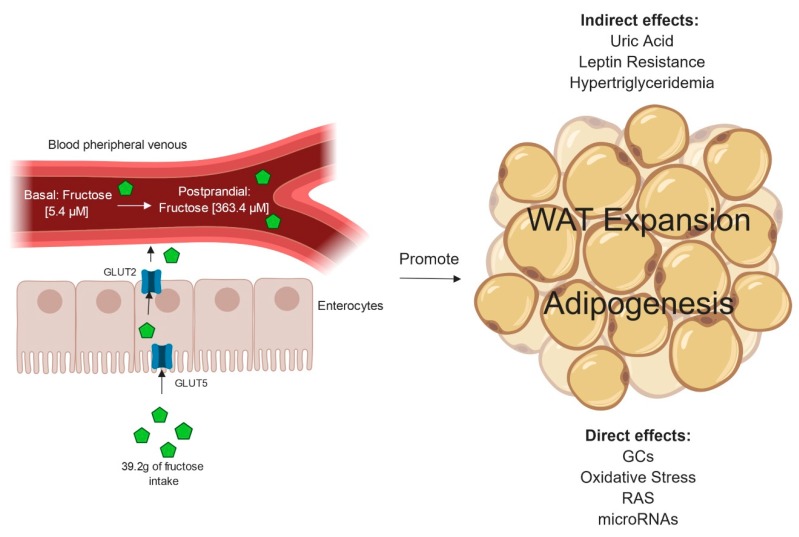
Chronic fructose intake induces white adipose tissue (WAT) expansion. Chronic fructose intake increases fructose concentration in the peripheral venous blood, which may reach WAT. Fructose may promote adipogenesis through direct mechanisms such as uric acid and leptin resistance or indirect mechanisms such as glucocorticoids action, oxidative stress, the renin–angiotensin system (RAS) and the induction of microRNAs. WAT: White adipose tissue; GCs: Glucocorticoids; RAS: Renin–angiotensin system. Created with Biorender.com.

Humans, as well as other species, can directly metabolize and absorb fructose from different sources. Sucrose and other sugars are metabolized into glucose and fructose in the intestine [16]. Fructose is absorbed in the brush border from the lower part of the duodenum and jejunum by the glucose transporter type 5 (GLUT5, also named SLC2A5). Meanwhile, GLUT2 is located basolaterally, and translocates fructose into the circulation [17] (Figure 1).

In mice, upon low fructose concentration intake, it may be metabolized in the splanchnic organs. However, fructose overload increases its circulatory concentrations [18]. In humans, Francey and collaborators used marked fructose and demonstrated that nearly 70% of fructose was directly metabolized by the liver [19], although other tissues such as WAT may metabolize the remaining amount. Also, chronic fructose intake can increase its absorption, promoting *GLUT5* gene overexpression in the intestine [20]. Thus, after high exposure, more fructose enters portal circulation and may escape the liver and enter into the systemic circulation.

The concentrations of systemic fructose, unlike glucose, fluctuate greatly, and very few studies have been conducted on portal circulation. In a study of healthy adults that ingested 30.4 g of fructose, 4.4 g of fructose reached the systemic circulation [19]. Another study demonstrated that intake of an HFCS-sweetened beverage containing 39.2 g of fructose and 28.8 g of glucose increased fructose concentration from 5.4 µM to 363.4 µM in peripheral venous blood [21]. Moreover, rats administered with 2 g/kg of a solution rich in fructose reached concentrations of 20 µM arterial and 146 µM peripheral blood. The same study found the highest fructose levels in the portal vein [22]. However, studies have shown that some amount of the fructose ingested may be excreted in the urine. Indeed, urinary fructose has been used as a reliable marker of sucrose and fructose intake. Campbell and collaborators demonstrated that 20.2 mg/L of urinary fructose was excreted by children and adolescents that consumed 75.7 g of fructose [23]. In another study, the fructose consumptions estimated in women and men were 117 g/day and 162 g/day, respectively. Meanwhile, the urinary fructose levels were 18.1 mg/day in men and 17.5 mg/day in women [24].

In addition, animal models have shown that some fructose may be reabsorbed in the kidney. Rats with chronic intake of 20% fructose (w/v) in drinking water demonstrated that proximal tubules reabsorbed fructose at a rate of 20 pmol/mm/min compared to 12.8 pmol/mm/min in control rats. The same study found that fructose consumption increased GLUT2, GLUT4 and GLUT5 expression in the proximal tubule [25]. In summary, this evidence clearly suggests that fructose reaches other metabolically active tissues, activating the expression of GLUT5 in tissues such as WAT.

## 3. Effects of Excessive Fructose Intake on WAT Accumulation

Chronic fructose intake has been demonstrated to promote WAT accumulation (Figure 1). Several studies have also indicated that high fructose intake in beverages induces WAT accumulation in human and rodent models. In adolescents, the consumption of 350 mL/day of HFCS-sweetened beverage induced insulin resistance and visceral WAT accumulation [26]. Also, adult subjects that consumed 75 g of fructose beverage had increased liver fat content, which positively correlated with WAT expansion [27]. On the contrary, opposite trends for visceral WAT accumulation were found in obese children given an isocaloric fructose restriction diet [28]. Furthermore, rats that consumed 60% (w/v) fructose in drinking water for nine weeks developed visceral WAT accumulation and elevated triglyceride levels [29]. Kovačević and collaborators found similar results in rats drinking a 10% fructose (w/v) solution for nine weeks [30]. Moreover, there are genes that modify how the organism responds to high fructose intake. Therefore, there is an association of at least 30 obesity-related genetic variants identified in genome-wide association studies (GWAS) with effects exacerbated by high SSB intake [31,32,33]. In sum, these studies indicate that excessive fructose consumption is an important inductor of WAT accumulation.

There are well-recognized indirect effects of high fructose intake promoting systemic factors that contribute to WAT expansion (Figure 1), including fructose-increased caloric intake mediated via leptin resistance [7] and the antagonism of glucagon-like peptide-1 receptor (GLP-1R) action in the brain [34], as well as hyperuricemia [1] and visceral WAT inflammation [35]. However, there is also evidence that suggests a more direct effect of fructose on WAT. Most of the evidence for the direct effect of fructose on WAT comes from studies made in vitro [15,36,37]. For example, GLUT5 expression and function play a role in adipocyte differentiation [15]. It has also been shown that fructose exposure increases GLUT5 expression in WAT [12]. These in vitro results suggest that fructose alone can induce adipogenesis. In adipocyte precursor cells (APCs) as well as in preadipocytes, fructose induces adipogenic programs. In rats, 10% fructose in drinking water increased the number of APCs and the percentage of differentiated adipocytes [14]. A few other studies have also shown that physiological levels of fructose (50–550 μM) can induce 3T3-L1 to differentiate [15]. In summary, fructose in the circulation can be responsible for the induction of WAT hyperplasia as well as adipocyte hypertrophy (Figure 1). Some of the mechanisms implicated are discussed further in this paper.

## 4. Fructose, Hypertriglyceridemia, Very-Low-Density Lipoproteins (VLDLs) and Adipogenesis 

High fructose intake has been associated with hepatic de novo lipogenesis (DNL), hypertriglyceridemia and increased visceral fat mass. In overweight and obese humans, in which 25% of their caloric requirements were given as glucose- or fructose-sweetened beverages for 10 weeks, only subjects receiving fructose showed a marked increment of plasma lipids and lipoprotein concentrations as well as postprandial triglycerides (TG). It was shown that both increased DNL induced by fructose and reduced clearance contributed to the rise in postprandial TG [8]. In addition, only fructose intake was associated with a significant increment of visceral WAT, despite the fact that both glucose and fructose groups gained comparable body weight at the end of the follow-up [8]. Moreover, in healthy adults, the intake of meals high in fructose also increased postprandial DNL without changes in fatty acid oxidation; such effects were better observed in women [38]. Fructose can induce DNL since it stimulates the carbohydrate-responsive element-binding protein (ChREBP) [39] via uric acid production and protein phosphatase 2A [40]. Fructose also activates the transcription factor sterol regulatory element-binding protein 1c (SREBP-1c), independent of insulin secretion via PPAR- coactivator 1 [36]. These transcriptor factors play an important role in the synthesis of fatty acid and triglycerides [41]. Thus, high fructose intake alters lipid metabolism, which increases VLDL lipoproteins and TG levels in circulation. In addition, it has been suggested that the hydrolysis of TG-rich lipoproteins is a major source of adipogenic free fatty acid, which may promote WAT expansion in cases of obesity [42].

**Figure 2:**
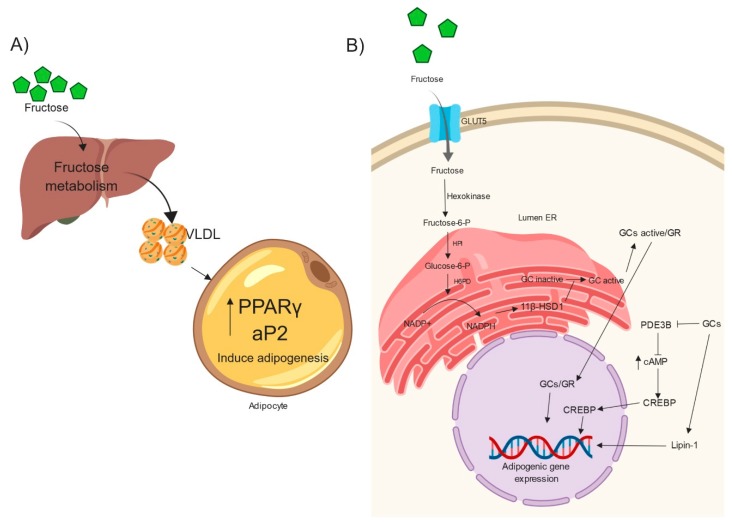
Fructose induces adipogenesis through very-low-density lipoprotein (VLDL) production and glucocorticoid (GC) signalling. (**A**) High fructose intake increases triglycerides and VLDL production, which induce adipogenesis by increasing PPARγ and aP2 expression. (**B**) Fructose metabolism in adipocytes promotes glucocorticoid activation by inducting 11β-HSD1. The effects of GCs in adipocyte precursors cells induce the expression of adipogenic genes through a few mechanisms. VLDL: Very-low-density lipoprotein; GCs: Glucocorticoids; GR: Glucocorticoid receptor; NADPH: Nicotinamide adenosine dinucleotide phosphate; 11β-HSD1: 11-beta hydroxysteroid dehydrogenase type 1; CREB: cAMP response element-binding protein; cAMP: Cyclic adenosine monophosphate; PDE3BB: Cyclic-nucleotide phosphodiesterase 3B; HPI: Hexose-phosphate isomerase; H6PD: Hexose-6-phosphate dehydrogenase. Created with Biorender.com.

High fructose intake promotes an increase in postprandial triglycerides levels with the VLDL lipoprotein fraction in plasma from rats [43] and humans [38]. VLDL promotes adipogenesis in an ApoE-dependent manner in 3T3-L1 cells [44]. In another study, Zhang and collaborators found similar results, using lipoproteins from hypertriglyceridemic individuals in 3T3-L1 cells by increasing aP2 and PPARϒ mRNA expression [45] (Figure 2a). Interestingly, in the same study, 3T3-L1 cells stimulated with lipoproteins and insulin showed higher induced adipogenic program compared to lipoprotein or insulin treatment alone [45]. Therefore, the increased VLDL levels by fructose metabolism in the liver may be an indirect effect of the induction of adipogenic program in WAT by high fructose intake (Figure 2a). 

## 5. Fructose Induces Adipogenesis by GC Action

In addition to their anti-inflammatory effects, GCs also play a role in the induction of WAT expansion. For example, in Cushing’s syndrome, hypercortisolemia increases intraabdominal and subcutaneous fat mass [46,47]. Thus, chronic GC treatments induce weight gain and WAT expansion [48]. It has been long recognized that obesity and obesogenic factors increase endogenous GCs. Fructose intake increases GCs, such as cortisol in human serum and WAT corticosterone levels in rats and mice [49]. It has been suggested that fructose intake may act as a modifier factor on inflammatory mechanisms in WAT.

In WAT cells, fructose has direct actions (Figure 2b)—it is first transported by GLUT5 and then metabolized to fructose-6-phosphate (F6P) by hexokinase. Subsequently, F6P is transported into the ER lumen, where there is a luminal hexose-phosphate isomerase (HPI) that converts F6P into glucose-6-phosphate, which is a substrate of the hexose-6-phosphate dehydrogenase (H6PDH) that generates the nicotinamide adenosine dinucleotide phosphate (NADPH) necessary for 11-beta hydroxysteroid dehydrogenase type 1 (11β-HSD1) oxidoreductase activity [50]. 11β-HSD1 is an NADPH-dependent enzyme that works as a tissue-specific amplifier of glucocorticoid action, which contributes significantly to the regulation of intracellular glucocorticoids levels through its activation from inactive 11-keto forms [51].

Fructose consumption increases active GC levels through 11β-HSD1 activity in rat WAT [52]. In accordance with this, an increase of corticosterone in WAT and plasma accompanied total WAT gain in rats fed with 10% fructose (w/v) in drinking water [53]. The authors also reported that this finding was due to the high activity/expression of H6PDH and 11β-HSD1 in epididymal WAT [53]. In vitro studies of fructose-induced adipogenesis in 3T3-L1 cells showed an increase in 11β-HSD1 expression, which promoted high endogenous GC levels [12]. Interestingly, glucose exposure did not induce 11β-HSD1 expression. These results support the idea that fructose probably causes WAT expansion by increasing 11β-HSD1 activity that promotes adipogenesis (Figure 2b).

As mentioned above, fructose increases active GCs in adipocytes. The glucocorticoid receptor (GR) mediates GC actions in these cells. GR is a nuclear receptor that recognizes GCs that, in their active form, are translocated to the nucleus [54]. Dexamethasone (DEX) is a synthetic GC that is used in the differentiation of 3T3-L1 preadipocytes to mature adipocytes. In this regard, DEX-bound GR accelerates adipogenesis, increasing the expression of adipogenic transcription factors such as C/EBPβ, C/EBPδ, Klf5, Klf9, C/EBPα and PPARγ [55]. Also, DEX downregulates cyclic-nucleotide phosphodiesterase 3B, which in turn elevates cellular cAMP and promotes the activating protein kinase A (PKA) [56]. In preadipocytes, elevated cAMP levels have been suggested to transcriptionally activate C/EBPβ by cAMP response element-binding protein (CREBP) [56]. In fructose-fed rats, GR activation is promoted, and this process may enhance adipogenesis by inducing adipogenic genes [57,58]. In this scenario, it could be speculated that GCs activate GR in preadipocytes upon fructose consumption, because fructose increases active GC levels in WAT. This process would generate the increase in the concentration of cAMP, which in turn activates CREBP. The induction of adipogenic genes by chronically increased levels of GCs by persistent fructose exposure could in part explain the enhanced adipogenesis.

Another molecule that plays a role in adipogenesis which is regulated by GCs is Lipin-1 (Figure 2B). This molecule participates in the accumulation of triglycerides in adipocytes. Lipin-1 is a phosphatidic acid phosphatase-1 that catalyzes the conversion of phosphatidate to diacylglycerol (the immediate precursor of triacylglycerol) [59]. In addition, DEX also induces an increase of Lipin-1 expression during the differentiation of 3T3-L1 preadipocytes [60]. In rats fed 60% fructose in drinking water, Lipin-1 expression was induced in the microsomal fraction from visceral WAT [29]. Also, Lipin-1 has been considered as an upstream factor that controls adipogenesis-related genes and regulates a positive feedback loop between PPARγ and C/EBPα in 3T3-L1 preadipocytes [61,62,63,64]. Conversely, in Lipin-1-deficient mice, a reduced WAT mass with an absence of mature adipocytes is found [64]. Therefore, it is possible that fructose consumption-induced GCs could promote adipocyte differentiation through the induction of Lipin-1 expression (Figure 2b). However, studies that explore the participation of Lipin-1 in adipogenesis induced by fructose are still lacking.

## 6. Reactive Oxygen Species and Fructose

Reactive oxygen species (ROS) are oxygen-derived small molecules, generated during physiological and pathological processes [65]. NADPH oxidases (NOX) and xanthine oxidase (XOR) as well as other enzymes participate in ROS production [66]. In addition, several studies indicate that ROS production induces signaling pathways regulating survival, apoptosis, proliferation, and differentiation factors [67,68]. In 3T3-L1 cells adipogenesis, ROS levels are increased. This extracellular oxidizing environmental status accelerates adipogenesis by increasing lipid accumulation and the expression of the adipogenic gene program [69]. Tormos and collaborators also found that lipogenesis and adipogenesis mitochondrial metabolism and ROS generation were dependent on mTORC1 signaling in human mesenchymal stem cells (MSC) [70]. Moreover, ROS inhibition leads to a decrease in the differentiation to mature adipocytes in 3T3-L1, and equine adipose-derived stromal cells [71] and antioxidant treatment showed similar results in 3T3-L1 cells [72]. These results suggest that an oxidizing environment produced by mitochondrial metabolism and ROS play a crucial role in promoting adipocyte differentiation.

**Figure 3:**
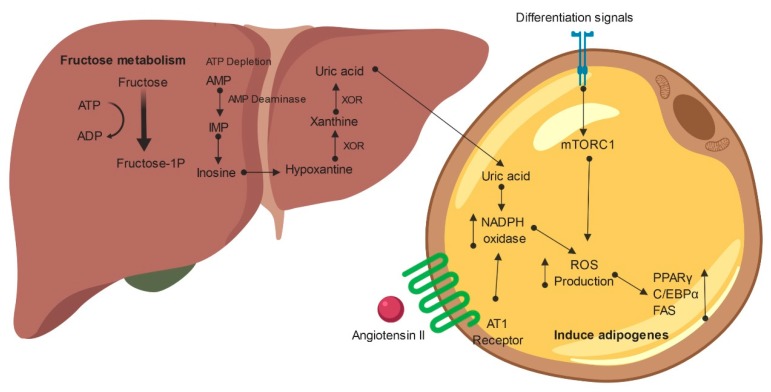
Fructose-induced reactive oxygen species (ROS) production promotes adipogenesis. Fructose metabolism induces adipogenesis through increasing ROS production. ROS may be induced by uric acid, NADPH oxidase activity, mTORC1 signaling and AT1 receptor signaling. ROS: Reactive oxygen species; NADPH: Nicotinamide adenosine dinucleotide phosphate; XOR: Xanthine oxidase; FAS: Fatty acid synthase; C/EBPα: CCAAT-enhancer-binding protein α; PPARϒ: Peroxisome proliferator-activated receptor ϒ. Created with Biorender.com.

Interestingly, fructose exposure increases ROS levels in several tissues such as WAT [73,74,75,76,77] (Figure 3). The oxidizing environment in WAT is a mechanism that sustains the obesity phenotype [78,79,80]. Moreover, fructose can induce more ROS production than glucose [81]. In human MSCs, fructose increases isoprostane and heme contents. Conversely, cobalt protoporphyrin, a heme oxygenase-1 (HO-1) inducer, decreases isoprostane and heme levels, thus also decreasing adipogenesis. In addition, HO-1 decreases the expression of β-catenin and Wnt10b (Wnt Family Member 10B), which are inhibitors of adipogenesis [82]. Therefore, some evidence indicates that ROS production may participate in the induction of adipogenesis by fructose consumption (Figure 3).

## 7. Uric Acid Resulting from Fructose Metabolism as a Mediator of Adipogenesis

Hyperuricemia (HU) is a common alteration that rises to more than 20% in some populations [83]. Obesity is closely related to hyperuricemia; in particular, visceral fat deposits are positively associated with increased uric acid production [84]. Therefore, the visceral adiposity index (which composes waist circumference, body mass index, triglycerides and high-density lipoprotein cholesterol) also correlates positively with HU in obese and even in metabolically healthy subjects [85,86].

Uric acid (UA) is a byproduct of fructose when it is phosphorylated by ketohexokinase (KHK). As KHK does not have a negative feedback mechanism, all the fructose presented is phosphorylated until ATP exhaustion [87] (Figure 3). As ADP and inorganic phosphorous accumulates inside the cell, the purine degradation pathway is activated, thus significantly increasing the intracellular uric acid concentrations [87]. This process occurs in hepatocytes, enterocytes, muscle cells and proximal tubule renal cells. In adipocytes, there is some indirect evidence of the activity of this pathway [88]. For example, in MSC-derived adipocytes, incubation with 500 mM fructose significantly increased xanthine oxidase expression as well as uric acid concentrations. Furthermore, adipogenesis was increased by 50%. Moreover, incubation with UA (5 mg/dL) also increased adipogenesis by 50%, augmented the number of large lipid droplets, and decreased the number of small droplets compared to the control. In addition, UA also increased the mRNA of C/EBPα and PPARγ, known mediators of adipocyte hyperplasia [88]. XOR is an enzyme that catalyzes the catabolism of purines such as xanthine and hypoxanthine to uric acid [89]. XOR expression in WAT tissue is abundant, and in obese mice, the expression and activity of this enzyme are higher [90]. Cultured adipocytes (primary mature adipocytes and 3T3-L1 cells) also produced and secreted uric acid into the culture medium. Those effects were inhibited by blocking XOR with febuxostat [90]. Thus, there is a possible link between fructose and the induction of adipogenesis via uric acid (Figure 3). 

Two mechanisms have been described so far to explain how UA may induce adipogenesis. (1) UA acts on adipocytes to increase ROS production through NADPH oxidase activation [88,91]. Nox4 is a major isoform of NADPH oxidase in adipocytes, which is activated upon UA exposure. The increase in intracellular oxidative stress induces the production and secretion of monocyte chemotactic protein 1 (MCP-1), a mediator of the proinflammatory state in adipocytes in obesity [91,92,93]. However, such an effect was blocked by Nox4. In obese mice, treatment with allopurinol reduced the production of MCP-1 in WAT, thus reducing macrophage infiltration. Kanda and collaborators also found that ROS were produced by Nox4-mediated adipocyte differentiation in 10T1/2 cells [94]. 2) UA upregulates the RAS in WAT. How the RAS is associated with adipogenesis is discussed in the next section.

## 8. RAS, Fructose and Adipogenesis

The RAS in visceral fat may play a role in WAT expansion by excessive fructose intake. Two studies showed that a fructose-rich diet increases Angiotensin II (Ang II) levels in plasma and WAT [95,96]. The RAS is also associated with increased visceral WAT [97]. Moreover, RAS inhibitors such as Temocapril, Olmesartan, Irbesartan and Aliskiren led to reduced Ang II levels in WAT as well as reduced visceral fat pad weight and adipocyte size in fructose-fed rats [98,99]. These studies suggest that the RAS may play a role in regulating visceral adiposity in fructose consumption.

On the other hand, the RAS may also contribute to ROS production in WAT. Ang II plays a role in ROS production through activating AT1 receptor, which promotes increased NOX expression [97,100] (Figure 3). Moreover, AT1 receptor is increased in WAT in fructose-fed rats [101]. As explained above, ROS production induces adipogenesis. Thus, the RAS may be in part responsible for ROS production and the induction of adipogenesis in WAT. However, it is necessary to clarify whether the RAS contributes to ROS production to induce adipogenesis in high fructose intake scenarios.

**Figure 4:**
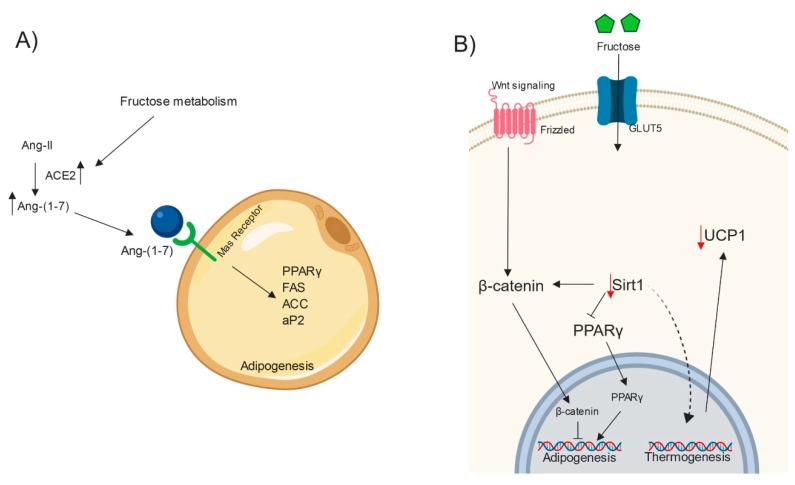
Fructose-induced adipogenesis through Ang-(1–7) and inhibition of thermogenesis. (**A**) Fructose metabolism promotes the conversion from Ang-II to Ang-(1–7) by increasing ACE2 activity. Mas receptor signaling induces the expression of adipogenic genes. (**B**) Sirt1 regulates the thermogenic program by inhibiting PPARγ and activating Wnt/β-catenin signalling. High fructose intake inhibits the thermogenic program by downregulating Sirt1 and UCP1 in WAT. ACE2: Angiotensin-converting enzyme 2; FAS: Fatty acid synthase; PPARϒ: Peroxisome proliferator-activated receptor ϒ; ACC: Acetyl-CoA carboxylase; aP2: Adipocyte protein 2; Sirt1: Sirtuin 1; UCP1: Uncoupling protein 1; Wnt: Wingless e Int. Created with Biorender.com.

Ang II undergoes hydrolysis by the angiotensin-converting enzyme (ACE) [102]; in particular, ACE2 is the crucial enzyme that generates Ang-(1–7) by the hydrolysis of Ang II [103]. Ang-(1–7) induces the oppose response compared to that of Ang II through its specific receptor (Mas) [104]. Both Ang-(1–7) and ACE2 were upregulated in WAT in sucrose-fed rats [105]. Ang-(1–7) and Mas receptor were also upregulated in 3T3-L1 cells [106]. However, Mas-KO 3T3-L1 cells showed decreased adipogenic markers, such as PPARγ, FAS, ACC, and aP2 [106]. As explained, Ang-(1–7) and Mas receptor may regulate adipocyte differentiation (Figure 4A). However, it is still necessary to study their participation in the adipogenesis process in fructose intake in humans.

## 9. Fructose, Sirtuin1, UCP1 and Thermogenesis

Sirtuin1 (Sirt1) is a member of the sirtuin family of NAD+-dependent protein deacetylases. Sirt1 participates as a cellular energy sensor and a mediator of caloric restriction. Also, Sirt1 regulates glucose and fat metabolism and plays a role in the adipogenic program [107,108,109,110]. Clinical studies have shown that mRNA expression of Sirt1 decreases in WAT in overweight and obese subjects [111,112]. In addition, in fructose-fed mice, Sirt1 protein levels in WAT are decreased [113]. However, Pektas and collaborators did not find changes in Sirt1 mRNA expression in WAT [77]. In mature adipocytes, Sirt1 promotes lipolysis and inhibits the adipogenic program in preadipocytes by repressing PPARγ activity [114]. Also, Sirt1 inhibits adipogenesis by activating Wnt/β-catenin signaling [110,115]. Moreover, delphinidin-3-O-β-glucoside, an anthocyanin with anti-adipogenic activity, promotes an increase in protein Sirt1 levels and downregulates the expression of adipogenic and lipogenic markers in 3T3-L1 preadipocytes [116]. Therefore, high fructose intake may induce adipogenesis by downregulating Sirt1 expression (Figure 4B). However, more studies are needed to elucidate whether fructose induces differentiation to adipocytes through direct regulation by Sirt1.

On the other hand, Sirt1 also plays a role in the induction of a thermogenic gene program in adipocytes. Sirt1 induces a brown adipocyte-like phenotype in white adipocytes by deacetylating PPARγ [117]. For example, in marrow adipose tissue (AT) from 129/Sv *Sirt1* haploin-sufficient (*Sirt1*^Δ/+^) mice, decreased expression of thermogenic genes was observed. In the same study, the inhibition of Sirt1 by EX527 downregulated *UCP1* in C3HT101/2 cells [118]. In fructose-fed mice, uncoupling protein 1 (*UCP1)* mRNA expression was decreased in subcutaneous WAT [119,120]. On the other hand, it has been demonstrated that fructose-fed rats experienced weight brown AT (BAT) gain [121,122], enlarging intracellular lipid droplets [123]. This lipid accumulation could be in part due to a decrease in UCP1 expression because, in mice fed a high-fat and high-fructose diet, *UCP1* mRNA expression in BAT was decreased [124]. These results indicate that high fructose intake may downregulate the thermogenic program in WAT (Figure 4B). Therefore, high fructose consumption may inhibit browning fate by promoting whitening fate in AT. 

## 10. MicroRNAs Implication in Fructose-Induced Adipogenesis

MicroRNAs (miRNAs) are involved in regulating several biological processes such as inflammation, metabolism, proliferation and differentiation, as well as adipogenesis [125]. miRNAs are short noncoding RNAs of ~22 nucleotides that bind to the 3′-UTR or 5′-UTR of target mRNA, leading to mRNA degradation or translational repression [125]. In humans, miRNAs regulate several genes involved in adipogenesis and obesity [126]. Also, miRNAs control the adipogenic gene expression program [127]. Few studies have established that the expression and function of several miRNAs may be involved in fructose overconsumption and lipogenic programs [128]. Moreover, to our knowledge, there are no studies investigating the role of miRNAs as well as other non-coding regulatory RNAs in fructose-induced adipogenesis. Therefore, in this section we provide a rationale for the implication of some miRNAs being related to adipogenesis caused by high fructose intake.

Some of the miRNAs that can be implicated in the induction of adipogenesis by high fructose intake are miR-206, miR-33, miR-122, miR-378a, miR-21, and miR-223. miR-206 negatively regulates adipogenesis. This miRNA acts on the c-Met/PI3K/Akt pathway in 3T3-L1 preadipocytes [129]. Fructose-fed rats showed decreased miR-206 expression in the renal cortex and podocytes [130]. On the other hand, mice deficient in miR-33 have greater WAT and adipocyte precursor cell numbers [131]. High fructose intake in these mice downregulates miR-33 expression in livers [128]. miR-122 is another candidate miRNA because it is downregulated in the liver in fructose-fed rats [132]. This miRNA suppresses PPARγ expression in 3T3-L1 preadipocytes [133]. Induction of miR-378a can also be involved. This miRNA promotes adipogenesis because it targets mitogen-activated protein kinase 1 [134]. Fructose also upregulates miR-378a in mouse livers [128]. Nevertheless, it is necessary to test if these miRNAs work similarly in WAT.

Considering that extracellular miRNA actions have been reported in several conditions, miR-21 and miR-223 were found to be upregulated in plasma microvesicles [135]. miR-223 promotes the differentiation of MSCs to adipocytes by regulating fibroblast growth factor receptor 2 (FGFR2) expression [136]. miR-21 also regulates adipogenesis in human WAT-derived MSCs. It modulates transforming growth factor beta (TGF-β) signaling [137] and activator protein 1 (AP-1) expression [138]. As explained above, fructose can modify miRNA expression; however, there are no studies demonstrating how fructose could modify either expression or the activity of miRNAs in adipogenesis. Studies explaining the role of circulating miRNAs in the condition of adipogenesis modulated by high fructose intake are needed.

## 11. Future Directions

- Studies that estimate the amount of fructose or its immediate metabolites that may enter into different types of WAT are needed.
- The acute metabolic and endocrine effects of high fructose intake on WAT from healthy and metabolically compromised individuals should be assessed.
- The role of fructose in the adipogenesis of newly discovered adipocyte subsets should be interesting to investigate.
- The reversibility of many high fructose intake-induced phenotypes should be studied.
- Studies with modest fructose intake, especially from sources other than processed food, such as fruits, are lacking.

## 12. Conclusions

In conclusion, high fructose intake has an important role in the current obesity epidemic. The fructose contained in beverages is likely the culprit leading to its excessive intake. The capacity of AT to cope with this nutrient overload is exceeded. From the studies using fructose in water or those that directly employed SSBs, it has been demonstrated that the high intake of this sugar may disturb several functions in adipocytes. Although there are many statements on how much total sugar intake is adequate (around 10% of the total caloric intake or less, according to the World Health Organization), there is no clarity in terms of free fructose intake.

## Abbreviations

HFCS	High-fructose corn syrup
WAT	White adipose tissue
SSBs	Sugar-sweetened beverages
GCs	Glucocorticoids
ROS	Reactive oxygen species
RAS	Renin–angiotensin system
miRNA	microRNA
GLUT5	Glucose transporter type 5
GWAS	Genome-wide association studies
GLP-1R	Glucagon-like peptide-1 receptor
APCs	Adipocyte precursor cells
F6P	Fructose-6-phosphate
NADPH	Nicotinamide adenosine dinucleotide phosphate
11β-HSD1	1β-Hydroxysteroid dehydrogenase type 1
H6PDH	Hexose-6-phosphate dehydrogenase
HPI	Hexose phosphate isomerase
DEX	Dexamethasone
GR	Glucocorticoid receptor
C/EBPα	CCAAT-enhancer-binding protein α
KLF	Krüppel-like factor
PPARγ	Peroxisome proliferator-activated receptor γ
PDE3B	Phosphodiesterase 3B
cAMP	Cyclic adenosine monophosphate
PKA	Protein kinase A
CREBP	cAMP response element-binding protein
NOX	NADPH oxidase
XOR	Xanthine oxidase
MSC	Mesenchymal stem cell
HO-1	Heme oxygenase-1
WNT10b	Wnt family member 10b
HU	Hyperuricemia
UA	Uric acid
KHK	Ketohexokinase
MCP-1	Monocyte chemotactic protein 1
Ang-II	Angiotensin II
ACE	Angiotensin-converting enzyme
FAS	Fatty acid synthase
ACC	Acetyl-CoA carboxylase
aP2	Adipocyte protein 2
FGFR2	Fibroblast growth factor receptor 2
TGF-β	Transforming growth factor β
AP-1	Activator protein 1
VLDL	Very-low-density lipoprotein
Sirt1	Sirtuin 1
UCP1	Uncoupling protein 1
G3P	Delphinidin-3-O-β-glucoside
BAT	Brown adipose tissue
Wnt	Wingless e Int

## References

[1] Johnson R.J., Sánchez-Lozada L.G., Andrews P., Lanaspa M.A. (2017). Perspective: A Historical and Scientific Perspective of Sugar and Its Relation with Obesity and Diabetes. Adv. Nutr..

[2] Malik V.S., Pan A., Willett W.C., Hu F.B. (2013). Sugar-sweetened beverages and weight gain in children and adults: A systematic review and meta-analysis. Am. J. Clin. Nutr..

[3] Yang Q., Zhang Z., Gregg E.W., Flanders W.D., Merritt R., Hu F.B. (2014). Added Sugar Intake and Cardiovascular Diseases Mortality Among US Adults. JAMA Intern Med..

[4] Chung M., Ma J., Patel K., Berger S., Lau J., Lichtenstein A.H. (2014). Fructose, high-fructose corn syrup, sucrose, and nonalcoholic fatty liver disease or indexes of liver health: A systematic review and meta-analysis. Am. J. Clin. Nutr..

[5] Jegatheesan P., De Bandt J.P. (2017). Fructose and NAFLD: The multifaceted aspects of fructose metabolism. Nutrients.

[6] Jensen T., Abdelmalek M.F., Sullivan S., Nadeau K.J., Green M., Roncal C., Nakagawa T., Kuwabara M., Sato Y., Kang D.-H. (2018). Fructose and sugar: A major mediator of non-alcoholic fatty liver disease. J. Hepatol..

[7] Shapiro A., Mu W., Roncal C., Cheng K.-Y., Johnson R.J., Scarpace P.J. (2008). Fructose-induced leptin resistance exacerbates weight gain in response to subsequent high-fat feeding. Am. J. Physiol. Integr. Comp. Physiol..

[8] Stanhope K.L., Schwarz J.M., Keim N.L., Griffen S.C., Bremer A.A., Graham J.L., Hatcher B., Cox C.L., Dyachenko A., Zhang W. (2009). Consuming fructose-sweetened, not glucose-sweetened, beverages increases visceral adiposity and lipids and decreases insulin sensitivity in overweight/obese humans. J. Clin. Investig..

[9] Ma J., McKeown N.M., Hwang S.-J., Hoffmann U., Jacques P.F., Fox C.S. (2016). Sugar-Sweetened Beverage Consumption Is Associated With Change of Visceral Adipose Tissue Over 6 Years of Follow-Up. Circulation.

[10] Litherland G.J., Hajduch E., Gould G.W., Hundal H.S. (2004). Fructose transport and metabolism in adipose tissue of Zucker rats: Diminished GLUT5 activity during obesity and insulin resistance. Mol. Cell. Biochem..

[11] Froesch E.R., Ginsberg J.L. (1962). Fructose metabolism of adipose tissue. I. Comparison of fructose and glucose metabolism in epididymal adipose tissue of normal rats. J. Biol. Chem..

[12] Legeza B., Balázs Z., Odermatt A. (2014). Fructose promotes the differentiation of 3T3-L1 adipocytes and accelerates lipid metabolism. FEBS Lett..

[13] Del Mar Romero M., Sabater D., Fernández-López J.A., Remesar X., Alemany M. (2015). Glycerol production from glucose and fructose by 3T3-L1 Cells: A mechanism of Adipocyte defense from excess substrate. PLoS ONE.

[14] Zubiría M., Alzamendi A., Moreno G., Rey M., Spinedi E., Giovambattista A. (2016). Long-Term Fructose Intake Increases Adipogenic Potential: Evidence of Direct Effects of Fructose on Adipocyte Precursor Cells. Nutrients.

[15] Du L., Heaney A.P. (2012). Regulation of adipose differentiation by fructose and GluT5. Mol. Endocrinol..

[16] Gericke B., Schecker N., Amiri M., Naim H.Y. (2017). Structure-function analysis of human sucrase-isomaltase identifies key residues required for catalytic activity. J. Biol. Chem..

[17] Jones H.F., Butler R.N., Brooks D.A. (2011). Intestinal fructose transport and malabsorption in humans. AJP Gastrointest. Liver Physiol..

[18] Jang C., Hui S., Lu W., Cowan A.J., Morscher R.J., Lee G., Liu W., Tesz G.J., Birnbaum M.J., Rabinowitz J.D. (2018). The Small Intestine Converts Dietary Fructose into Glucose and Organic Acids. Cell. Metab..

[19] Francey C., Cros J., Rosset R., Crézé C., Rey V., Stefanoni N., Schneiter P., Tappy L., Seyssel K. (2019). The extra-splanchnic fructose escape after ingestion of a fructose–glucose drink: An exploratory study in healthy humans using a dual fructose isotope method. Clin. Nutr. ESPEN.

[20] Ferraris R.P., Choe J., Patel C.R. (2018). Intestinal Absorption of Fructose. Annu. Rev. Nutr..

[21] Le M.T., Frye R.F., Rivard C.J., Cheng J., McFann K.K., Segal M.S., Johnson R.J., Johnson J.A. (2012). Effects of high-fructose corn syrup and sucrose on the pharmacokinetics of fructose and acute metabolic and hemodynamic responses in healthy subjects. Metabolism.

[22] Sugimoto K., Hosotani T., Kawasaki T., Nakagawa K., Hayashi S., Nakano Y., Inui H., Yamanouchi T. (2010). Eucalyptus leaf extract suppresses the postprandial elevation of portal, cardiac and peripheral fructose concentrations after sucrose ingestion in rats. J. Clin. Biochem. Nutr..

[23] Intemann T., Pigeot I., De Henauw S., Eiben G., Lissner L., Krogh V., Dereń K., Molnár D., Moreno L.A., Russo P. (2018). Urinary sucrose and fructose to validate self-reported sugar intake in children and adolescents: Results from the I.Family study. Eur. J. Nutr..

[24] Campbell R., Tasevska N., Jackson K.G., Sagi-Kiss V., di Paolo N., Mindell J.S., Lister S.J., Khaw K.-T., Kuhnle G.G.C. (2017). Association between urinary biomarkers of total sugars and sucrose intake and BMI in a cross-sectional study. BioRxiv.

[25] Gonzalez-Vicente A., Cabral P.D., Hong N.J., Asirwatham J., Saez F., Garvin J.L. (2018). Fructose Reabsorption By Rat Proximal Tubules: Role of Sodium-Linked Cotransporters and the Effect of Dietary Fructose. Am. J. Physiol. Ren. Physiol..

[26] Lin W.T., Chan T.F., Huang H.L., Lee C.Y., Tsai S., Wu P.W., Yang Y.C., Wang T.N., Lee C.H. (2016). Fructose-Rich Beverage Intake and Central Adiposity, Uric Acid, and Pediatric Insulin Resistance. J. Pediatr..

[27] Taskinen M.-R., Söderlund S., Bogl L.H., Hakkarainen A., Matikainen N., Pietiläinen K.H., Räsänen S., Lundbom N., Björnson E., Eliasson B. (2017). Adverse effects of fructose on cardiometabolic risk factors and hepatic lipid metabolism in subjects with abdominal obesity. J. Intern. Med..

[28] Schwarz J.-M., Noworolski S.M., Erkin-Cakmak A., Korn N.J., Wen M.J., Tai V.W., Jones G.M., Palii S.P., Velasco-Alin M., Pan K. (2017). Effects of Dietary Fructose Restriction on Liver Fat, De Novo Lipogenesis, and Insulin Kinetics in Children With Obesity. Gastroenterology.

[29] Bursać B.N., Vasiljević A.D., Nestorović N.M., Veličković N.A., Vojnović Milutinović D.D., Matić G.M., Djordjevic A.D. (2014). High-fructose diet leads to visceral adiposity and hypothalamic leptin resistance in male rats—Do glucocorticoids play a role?. J. Nutr. Biochem..

[30] Kovačević S., Nestorov J., Matić G., Elaković I. (2014). Dietary fructose-related adiposity and glucocorticoid receptor function in visceral adipose tissue of female rats. Eur. J. Nutr..

[31] Qi Q., Chu A.Y., Kang J.H., Jensen M.K., Curhan G.C., Pasquale L.R., Ridker P.M., Hunter D.J., Willett W.C., Rimm E.B. (2012). Sugar-Sweetened Beverages and Genetic Risk of Obesity. New Engl. J. Med..

[32] Brunkwall L., Chen Y., Hindy G., Rukh G., Ericson U., Barroso I., Johansson I., Franks P.W., Orho-Melander M., Renström F. (2016). Sugar-sweetened beverage consumption and genetic predisposition to obesity in 2 Swedish cohorts. Am. J. Clin. Nutr..

[33] Olsen N.J., Ängquist L., Larsen S.C., Linneberg A., Skaaby T., Husemoen L.L.N., Toft U., Tjønneland A., Halkjær J., Hansen T. (2016). Interactions between genetic variants associated with adiposity traits and soft drinks in relation to longitudinal changes in body weight and waist circumference. Am. J. Clin. Nutr..

[34] Burmeister M.A., Ayala J., Drucker D.J., Ayala J.E. (2013). Central glucagon-like peptide 1 receptor-induced anorexia requires glucose metabolism-mediated suppression of AMPK and is impaired by central fructose. Am. J. Physiol. Endocrinol. Metab..

[35] Marek G., Pannu V., Shanmugham P., Pancione B., Mascia D., Crosson S., Ishimoto T., Sautin Y.Y. (2015). Adiponectin resistance and proinflammatory changes in the visceral adipose tissue induced by fructose consumption via ketohexokinase-dependent pathway. Diabetes.

[36] Pinnick K., Cornfield T., Green C., Hodson L. (2018). Fructose is metabolised by human subcutaneous adipocytes and can be used as a substrate for *de novo lipogenesis*. Endocr. Abstr..

[37] Robubi A., Huber K.R., Krugluger W. (2014). Extra fructose in the growth medium fuels lipogenesis of adipocytes. J. Obes..

[38] Low W., Cornfield T., Charlton C., Tomlinson J., Hodson L., Low W.S. (2018). Sex Differences in Hepatic De Novo Lipogenesis with Acute Fructose Feeding. Nutrients.

[39] Pinnick K.E., Hodson L. (2019). Challenging metabolic tissues with fructose: Tissue-specific and sex-specific responses. J. Physiol..

[40] Sanchez-Lozada L.G., Andres-Hernando A., Garcia-Arroyo F.E., Cicerchi C., Li N., Kuwabara M., Roncal-Jimenez C.A., Johnson R.J., Lanaspa M.A. (2019). Uric acid activates aldose reductase and the polyol pathway for endogenous fructose and fat production causing development of fatty liver in rats. J. Biol. Chem..

[41] Karagianni P., Talianidis I. (2015). Transcription factor networks regulating hepatic fatty acid metabolism. Biochim. Biophys. Acta Mol. Cell Biol. Lipids.

[42] Gonzales A.M., Orlando R.A. (2007). Role of adipocyte-derived lipoprotein lipase in adipocyte hypertrophy. Nutr. Metab..

[43] Maithilikarpagaselvi N., Sridhar M.G., Swaminathan R.P., Sripradha R., Badhe B. (2016). Curcumin inhibits hyperlipidemia and hepatic fat accumulation in high-fructose-fed male Wistar rats. Pharm. Biol..

[44] Chiba T., Nakazawa T., Yui K., Kaneko E., Shimokado K. (2003). VLDL induces adipocyte differentiation in ApoE-dependent manner. Arterioscler. Thromb. Vasc. Biol..

[45] Zhang M., Li Y., Wei X., Tian F., Ouyang F., Zhao S., Liu L. (2017). Indispensable role of lipoprotein bound-ApoE in adipogenesis and endocytosis induced by postprandial TRL. Biochem. Biophys. Res. Commun..

[46] Ferraù F., Korbonits M. (2015). Metabolic comorbidities in Cushing’s syndrome. Eur. J. Endocrinol..

[47] Delivanis D.A., Iñiguez-Ariza N.M., Zeb M.H., Moynagh M.R., Takahashi N., McKenzie T.J., Thomas M.A., Gogos C., Young W.F., Bancos I. (2018). Impact of hypercortisolism on skeletal muscle mass and adipose tissue mass in patients with adrenal adenomas. Clin. Endocrinol..

[48] Do T.T.H., Marie G., Héloïse D., Guillaume D., Marthe M., Bruno F., Buyse M. (2019). Glucocorticoid-induced insulin resistance is related to macrophage visceral adipose tissue infiltration. J. Steroid Biochem. Mol. Biol..

[49] DiNicolantonio J.J., Mehta V., Onkaramurthy N., O’Keefe J.H. (2018). Fructose-induced inflammation and increased cortisol: A new mechanism for how sugar induces visceral adiposity. Prog. Cardiovasc. Dis..

[50] Senesi S., Legeza B., Balázs Z., Csala M., Marcolongo P., Kereszturi É., Szelényi P., Egger C., Fulceri R., Mandl J. (2010). Contribution of Fructose-6-Phosphate to Glucocorticoid Activation in the Endoplasmic Reticulum: Possible Implication in the Metabolic Syndrome. Endocrinology.

[51] Walker B.R., Andrew R. (2006). Tissue Production of Cortisol by 11β-Hydroxysteroid Dehydrogenase Type 1 and Metabolic Disease. Ann. NY Acad. Sci..

[52] London E., Castonguay T.W. (2011). High Fructose Diets Increase 11β-Hydroxysteroid Dehydrogenase Type 1 in Liver and Visceral Adipose in Rats Within 24-h Exposure. Obesity.

[53] Prince P.D., Santander Y.A., Gerez E.M., Höcht C., Polizio A.H., Mayer M.A., Taira C.A., Fraga C.G., Galleano M., Carranza A. (2017). Fructose increases corticosterone production in association with NADPH metabolism alterations in rat epididymal white adipose tissue. J. Nutr. Biochem..

[54] Park Y.-K., Ge K. (2017). Glucocorticoid Receptor Accelerates, but Is Dispensable for, Adipogenesis. Mol. Cell. Biol..

[55] Xu C., He J., Jiang H., Zu L., Zhai W., Pu S., Xu G. (2009). Direct Effect of Glucocorticoids on Lipolysis in Adipocytes. Mol. Endocrinol..

[56] Niehof M., Manns M.P., Trautwein C. (1997). CREB controls LAP/C/EBP beta transcription. Mol. Cell. Biol..

[57] Bursać B.N., Djordjevic A.D., Vasiljević A.D., Milutinović D.D.V., Veličković N.A., Nestorović N.M., Matic G.M. (2013). Fructose consumption enhances glucocorticoid action in rat visceral adipose tissue. J. Nutr. Biochem..

[58] Lefterova M.I., Haakonsson A.K., Lazar M.A., Mandrup S. (2014). PPARγ and the global map of adipogenesis and beyond. Trends Endocrinol. Metab..

[59] Donkor J., Sariahmetoglu M., Dewald J., Brindley D.N., Reue K. (2006). Three Mammalian Lipins Act as Phosphatidate Phosphatases with Distinct Tissue Expression Patterns. J. Biol. Chem..

[60] Zhang P., O’Loughlin L., Brindley D.N., Reue K. (2008). Regulation of lipin-1 gene expression by glucocorticoids during adipogenesis. J. Lipid Res..

[61] Hu M., Wang F., Li X., Rogers C.Q., Liang X., Finck B.N., Mitra M.S., Zhang R., Mitchell D.A., You M. (2012). Regulation of hepatic lipin-1 by ethanol: Role of AMP-activated protein kinase/sterol regulatory element-binding protein 1 signaling in mice. Hepatology.

[62] Koh Y.-K., Lee M.-Y., Kim J.-W., Kim M., Moon J.-S., Lee Y.-J., Ahn Y.-H., Kim K.-S. (2008). Lipin1 Is a Key Factor for the Maturation and Maintenance of Adipocytes in the Regulatory Network with CCAAT/Enhancer-binding Protein α and Peroxisome Proliferator-activated Receptor γ_2_. J. Biol. Chem..

[63] Tang L.Y., Chen Y., Rui B.B., Hu C.M. (2016). Resveratrol ameliorates lipid accumulation in HepG2 cells, associated with down-regulation of lipin1 expression. Can. J. Physiol. Pharmacol..

[64] Csaki L.S., Reue K. (2010). Lipins: Multifunctional Lipid Metabolism Proteins. Annu. Rev. Nutr..

[65] Soberman R.J. (2003). Series Introduction: The expanding network of redox signaling: New observations, complexities, and perspectives. J. Clin. Investig..

[66] Braunersreuther V., Jaquet V. (2012). Reactive Oxygen Species in Myocardial Reperfusion Injury: From Physiopathology to Therapeutic Approaches. Curr. Pharm. Biotechnol..

[67] Coso S., Harrison I., Harrison C.B., Vinh A., Sobey C.G., Drummond G.R., Williams E.D., Selemidis S. (2012). NADPH Oxidases as Regulators of Tumor Angiogenesis: Current and Emerging Concepts. Antioxid. Redox Signal..

[68] Storz P. (2011). Forkhead Homeobox Type O Transcription Factors in the Responses to Oxidative Stress. Antioxid. Redox Signal..

[69] Imhoff B.R., Hansen J.M. (2010). Extracellular redox environments regulate adipocyte differentiation. Differentiation.

[70] Tormos K.V., Anso E., Hamanaka R.B., Eisenbart J., Joseph J., Kalyanaraman B., Chandel N.S. (2011). Mitochondrial Complex III ROS Regulate Adipocyte Differentiation. Cell Metab..

[71] Han J., Choi H.Y., Dayem A.A., Kim K., Yang G., Won J., Do S.H., Kim J.-H., Jeong K.-S., Cho S.-G. (2017). Regulation of Adipogenesis Through Differential Modulation of ROS and Kinase Signaling Pathways by 3,4’-Dihydroxyflavone Treatment. J. Cell. Biochem..

[72] Lee Y.-J., Seo M.-J., Lee O.-H., Kim K.-J., Lee B.-Y. (2017). Hesperetin inhibits lipid accumulation and ROS production during adipocyte differentiation in 3T3-L1 cells. J. Food Biochem..

[73] Delbosc S., Paizanis E., Magous R., Araiz C., Dimo T., Cristol J.-P., Cros G., Azay J. (2005). Involvement of oxidative stress and NADPH oxidase activation in the development of cardiovascular complications in a model of insulin resistance, the fructose-fed rat. Atherosclerosis.

[74] Jaiswal N., Maurya C.K., Pandey J., Rai A.K., Tamrakar A.K. (2015). Fructose-induced ROS generation impairs glucose utilization in L6 skeletal muscle cells. Free. Radic. Res..

[75] Crescenzo R., Bianco F., Coppola P., Mazzoli A., Valiante S., Liverini G., Lossa S. (2014). Adipose tissue remodeling in rats exhibiting fructose-induced obesity. Eur. J. Nutr..

[76] Bratoeva K., Radanova M., Merdzhanova A., Donev I. (2017). Protective role of S-Adenosylmethionine against fructose-induced oxidative damage in obesity. J. Mind Med. Sci..

[77] Pektas M.B., Koca H.B., Sadi G., Akar F. (2016). Dietary Fructose Activates Insulin Signaling and Inflammation in Adipose Tissue: Modulatory Role of Resveratrol. BioMed Res. Int..

[78] Crujeiras A.B., Díaz-Lagares A., Carreira M.C., Amil M., Casanueva F.F. (2013). Oxidative stress associated to dysfunctional adipose tissue: A potential link between obesity, type 2 diabetes mellitus and breast cancer. Free Radic. Res..

[79] Lafontan M. (2014). Adipose tissue and adipocyte dysregulation. Diabetes Metab..

[80] Zhang X., Zhang J.-H., Chen X.-Y., Hu Q.-H., Wang M.-X., Jin R., Zhang Q.-Y., Wang W., Wang R., Kang L.-L. (2015). Reactive oxygen species-induced TXNIP drives fructose-mediated hepatic inflammation and lipid accumulation through NLRP3 inflammasome activation. Antioxid. Redox Signal..

[81] Araoye E., Ckless K. (2016). Effects of High Fructose/Glucose on Nlrp3/Il1β Inflammatory Pathway. J. Young Investig..

[82] Khitan Z., Harsh M., Sodhi K., Shapiro J.I., Abraham N.G. (2014). HO-1 Upregulation Attenuates Adipocyte Dysfunction, Obesity, and Isoprostane Levels in Mice Fed High Fructose Diets. J. Nutr. Metab..

[83] Zhu Y., Pandya B.J., Choi H.K. (2012). Comorbidities of Gout and Hyperuricemia in the US General Population: NHANES 2007–2008. Am. J. Med..

[84] Matsuura F., Yamashita S., Nakamura T., Nishida M., Nozaki S., Funahashi T., Matsuzawa Y. (1998). Effect of visceral fat accumulation on uric acid metabolism in male obese subjects: Visceral fat obesity is linked more closely to overproduction of uric acid than subcutaneous fat obesity. Metabolism.

[85] Gu D., Ding Y., Zhao Y., Miao S., Qu Q. (2018). Positively increased visceral adiposity index in hyperuricemia free of metabolic syndrome. Lipids Health Dis..

[86] Dong H., Xu Y., Zhang X., Tian S. (2017). Visceral adiposity index is strongly associated with hyperuricemia independently of metabolic health and obesity phenotypes. Sci. Rep..

[87] Lanaspa M.A., Tapia E., Soto V., Sautin Y., Sánchez-Lozada L.G. (2011). Uric Acid and Fructose: Potential Biological Mechanisms. Semin. Nephrol..

[88] Sodhi K., Hilgefort J., Banks G., Gilliam C., Stevens S., Ansinelli H.A., Getty M., Abraham N.G., Shapiro J.I., Khitan Z. (2016). Uric Acid-Induced Adipocyte Dysfunction Is Attenuated by HO-1 Upregulation: Potential Role of Antioxidant Therapy to Target Obesity. Stem Cells Int..

[89] Berry C.E., Hare J.M. (2004). Xanthine oxidoreductase and cardiovascular disease: Molecular mechanisms and pathophysiological implications. J. Physiol..

[90] Tsushima Y., Nishizawa H., Tochino Y., Nakatsuji H., Sekimoto R., Nagao H., Shirakura T., Kato K., Imaizumi K., Takahashi H. (2013). Uric acid secretion from adipose tissue and its increase in obesity. J. Biol. Chem..

[91] Sautin Y.Y., Nakagawa T., Zharikov S., Johnson R.J. (2007). Adverse effects of the classic antioxidant uric acid in adipocytes: NADPH oxidase-mediated oxidative/nitrosative stress. Am. J. Physiol. Cell Physiol..

[92] Baldwin W., McRae S., Marek G., Wymer D., Pannu V., Baylis C., Johnson R.J., Sautin Y.Y. (2011). Hyperuricemia as a Mediator of the Proinflammatory Endocrine Imbalance in the Adipose Tissue in a Murine Model of the Metabolic Syndrome. Diabetes.

[93] Henriksen E.J., Diamond-Stanic M.K., Marchionne E.M. (2011). Oxidative stress and the etiology of insulin resistance and type 2 diabetes. Free Radic. Biol. Med..

[94] Kanda Y., Hinata T., Kang S.W., Watanabe Y. (2011). Reactive oxygen species mediate adipocyte differentiation in mesenchymal stem cells. Life Sci..

[95] Giani J.F., Muñoz M.C., Mayer M.A., Veiras L.C., Arranz C., Taira C.A., Turyn D., Toblli J.E., Dominici F.P. (2010). Angiotensin-(1–7) improves cardiac remodeling and inhibits growth-promoting pathways in the heart of fructose-fed rats. Am. J. Physiol. Heart Circ. Physiol..

[96] Tran L.T., Yuen V.G., McNeill J.H. (2009). The fructose-fed rat: A review on the mechanisms of fructose-induced insulin resistance and hypertension. Mol. Cell. Biochem..

[97] Chou C.-L., Lin H., Chen J.-S., Fang T.-C. (2017). Renin inhibition improves metabolic syndrome, and reduces angiotensin II levels and oxidative stress in visceral fat tissues in fructose-fed rats. PLoS ONE.

[98] Furuhashi M., Ura N., Takizawa H., Yoshida D., Moniwa N., Murakami H., Higashiura K., Shimamoto K. (2004). Blockade of the renin-angiotensin system decreases adipocyte size with improvement in insulin sensitivity. J. Hypertens..

[99] Muñoz M.C., Giani J.F., Dominici F.P., Turyn D., Toblli J.E. (2009). Long-term treatment with an angiotensin II receptor blocker decreases adipocyte size and improves insulin signaling in obese Zucker rats. J. Hypertens..

[100] Shinozaki K., Ayajiki K., Nishio Y., Sugaya T., Kashiwagi A., Okamura T. (2004). Evidence for a Causal Role of the Renin-Angiotensin System in Vascular Dysfunction Associated With Insulin Resistance. Hypertension.

[101] Magliano D.C., Penna-de-Carvalho A., Vazquez-Carrera M., Mandarim-de-Lacerda C.A., Aguila M.B. (2015). Short-term administration of GW501516 improves inflammatory state in white adipose tissue and liver damage in high-fructose-fed mice through modulation of the renin-angiotensin system. Endocrine.

[102] Harrison-Bernard L.M. (2009). The renal renin-angiotensin system. Adv. Physiol. Educ..

[103] Donoghue M., Hsieh F., Baronas E., Godbout K., Gosselin M., Stagliano N., Donovan M., Woolf B., Robison K., Jeyaseelan R. (2000). A novel angiotensin-converting enzyme-related carboxypeptidase (ACE2) converts angiotensin I to angiotensin 1–9. Circ. Res..

[104] Santos R.A.S., Ferreira A.J., Simões e Silva A.C. (2008). Recent advances in the angiotensin-converting enzyme 2—Angiotensin(1–7)—Mas axis. Exp. Physiol..

[105] Coelho M.S., Lopes K.L., Freitas R de A., de Oliveira-Sales E.B., Bergasmaschi C.T., Campos R.R., Casarini D.E., Carmona A.K., da SilvaAraújo M., Heimann J.C. (2010). High sucrose intake in rats is associated with increased ACE2 and angiotensin—(1–7) levels in the adipose tissue. Regul Pept..

[106] Than A., Leow M.K.-S., Chen P. (2013). Control of adipogenesis by the autocrine interplays between angiotensin 1–7/Mas receptor and angiotensin II/AT1 receptor signaling pathways. J. Biol. Chem..

[107] Guarente L., Picard F. (2005). Calorie restriction—The SIR2 connection. Cell.

[108] Bordone L., Cohen D., Robinson A., Motta M.C., Van Veen E., Czopik A., Steele A.D., Crowe H., Marmor S., Luo J. (2007). SIRT1 transgenic mice show phenotypes resembling calorie restriction. Aging Cell.

[109] Haigis M.C., Guarente L.P. (2006). Mammalian sirtuins—Emerging roles in physiology, aging, and calorie restriction. Genes Dev..

[110] Zhou Y., Song T., Peng J., Zhou Z., Wei H., Zhou R., Jiang S., Peng J. (2016). SIRT1 suppresses adipogenesis by activating Wnt/B-catenin signaling in vivo and in vitro. Oncotarget.

[111] Martínez-Jiménez V., Cortez-Espinosa N., Rodríguez-Varela E., Vega-Cárdenas M., Briones-Espinoza M., Ruíz-Rodríguez V.M., López-López N., Briseño-Medina A., Turiján-Espinoza E., Portales-Pérez D.P. (2019). Altered levels of sirtuin genes (SIRT1, SIRT2, SIRT3 and SIRT6) and their target genes in adipose tissue from individual with obesity. Diabetes Metab. Syndr. Clin. Res. Rev..

[112] Kurylowicz A., Owczarz M., Polosak J., Jonas M.I., Lisik W., Jonas M., Chmura A., Kuznicka M.P. (2016). SIRT1 and SIRT7 expression in adipose tissues of obese and normal-weight individuals is regulated by microRNAs but not by methylation status. Int. J. Obes..

[113] Gillum M.P., Kotas M.E., Erion D.M., Kursawe R., Chatterjee P., Nead K.T., Muise E.S., Hsiao J.J., Frederick D.W., Yonemitsu S. (2011). SirT1 regulates adipose tissue inflammation. Diabetes.

[114] Picard F., Kurtev M., Chung N., Topark-Ngarm A., Senawong T., Machado de Oliveira R., Leid M., McBurney M.W., Guarente L. (2004). SIRT1 promotes fat mobilization in white adipocytes by repressing PPAR-γ. Nature.

[115] Zhou Y., Zhou Z., Zhang W., Hu X., Wei H., Peng J., Liang S. (2015). SIRT1 inhibits adipogenesis and promotes myogenic differentiation in C3H10T1/2 pluripotent cells by regulating Wnt signaling. Cell Biosci..

[116] Park M., Sharma A., Lee H.-J., Park M., Sharma A., Lee H.-J. (2019). Anti-Adipogenic Effects of Delphinidin-3-O-β-Glucoside in 3T3-L1 Preadipocytes and Primary White Adipocytes. Molecules.

[117] Qiang L., Wang L., Kon N., Zhao W., Lee S., Zhang Y., Rosenbaum M., Zhao Y., Gu W., Farmer S.R. (2012). Brown remodeling of white adipose tissue by SirT1-dependent deacetylation of Pparγ. Cell.

[118] Artsi H., Gurt I., El-Haj M., Müller R., Kuhn G.A., Ben Shalom G., Cohen-Kfir E., Abramowitz E., Kandel L., Safran O. (2019). Sirt1 Promotes a Thermogenic Gene Program in Bone Marrow Adipocytes: From Mice to (Wo)Men. Front. Endocrinol..

[119] Dobner J., Ress C., Rufinatscha K., Salzmann K., Salvenmoser W., Folie S., Wieser V., Moser P., Weiss G., Goebel G. (2017). Fat-enriched rather than high-fructose diets promote whitening of adipose tissue in a sex-dependent manner. J. Nutr. Biochem..

[120] Bargut T.C.L., Martins F.F., Santos L.P., Aguila M.B., Mandarim-de-Lacerda C.A. (2019). Administration of eicosapentaenoic and docosahexaenoic acids may improve the remodeling and browning in subcutaneous white adipose tissue and thermogenic markers in brown adipose tissue in mice. Mol. Cell. Endocrinol..

[121] Kanarek R.B., Orthen-Gambill N. (1982). Differential Effects of Sucrose, Fructose and Glucose on Carbohydrate-Induced Obesity in Rats. J. Nutr..

[122] Jatkar A., Kurland I.J., Judex S. (2017). Diets High in Fat or Fructose Differentially Modulate Bone Health and Lipid Metabolism. Calcif. Tissue Int..

[123] Machado T.Q., Pereira-Silva D.C., Gonçalves L.F., Fernandes-Santos C. (2019). Brown Adipose Tissue Remodeling Precedes Cardiometabolic Abnormalities Independent of Overweight in Fructose-Feed Mice. BioRxiv.

[124] Liu Z., Qiao Q., Sun Y., Chen Y., Ren B., Liu X. (2017). Sesamol ameliorates diet-induced obesity in C57BL/6J mice and suppresses adipogenesis in 3T3-L1 cells via regulating mitochondria-lipid metabolism. Mol. Nutr. Food Res..

[125] Gebert L.F.R., MacRae I.J. (2019). Regulation of microRNA function in animals. Nat. Rev. Mol. Cell Biol..

[126] Shi C., Zhang M., Tong M., Yang L., Pang L., Chen L., Xu G., Chi X., Hong Q., Ni Y. (2015). miR-148a is Associated with Obesity and Modulates Adipocyte Differentiation of Mesenchymal Stem Cells through Wnt Signaling. Sci. Rep..

[127] Arner P., Kulyté A. (2015). MicroRNA regulatory networks in human adipose tissue and obesity. Nat. Rev. Endocrinol..

[128] Sud N., Zhang H., Pan K., Cheng X., Cui J., Su Q. (2017). Aberrant expression of microRNA induced by high-fructose diet: Implications in the pathogenesis of hyperlipidemia and hepatic insulin resistance. J. Nutr. Biochem..

[129] Tang R., Ma F., Li W., Ouyang S., Liu Z., Wu J. (2017). miR-206-3p Inhibits 3T3-L1 Cell Adipogenesis via the c-Met/PI3K/Akt Pathway. Int. J. Mol. Sci..

[130] Ding X.Q., Gu T.T., Wang W., Song L., Chen T.Y., Xue Q.C., Zhou F., Li J.-M., Kong L.-D. (2015). Curcumin protects against fructose-induced podocyte insulin signaling impairment through upregulation of miR-206. Mol. Nutr. Food Res..

[131] Price N.L., Singh A.K., Rotllan N., Goedeke L., Wing A., Canfrán-Duque A., Diaz-Ruiz A., Araldi E., Baldán A., Camporez J.-P. (2018). Genetic Ablation of miR-33 Increases Food Intake, Enhances Adipose Tissue Expansion, and Promotes Obesity and Insulin Resistance. Cell Rep..

[132] Alisi A., Da Sacco L., Bruscalupi G., Piemonte F., Panera N., De Vito R., Leoni S., Bottazzo G.F., Masotti A., Nobili V. (2011). Mirnome analysis reveals novel molecular determinants in the pathogenesis of diet-induced nonalcoholic fatty liver disease. Lab. Investig..

[133] Liao C.H., Wang C.Y., Liu K.H., Liu Y.Y., Wen M.S., Yeh T.S. (2018). MiR-122 marks the differences between subcutaneous and visceral adipose tissues and associates with the outcome of bariatric surgery. Obes. Res. Clin. Pract..

[134] Huang N., Wang J., Xie W., Lyu Q., Wu J., He J., Qiu W., Xu N., Zhang Y. (2015). MiR-378a-3p enhances adipogenesis by targeting mitogen-activated protein kinase 1. Biochem. Biophys. Res. Commun..

[135] Brianza-Padilla M., Carbó R., Arana J.C., Vázquez-Palacios G., Ballinas-Verdugo M.A., Cardoso-Saldaña G.C., Palacio A.G., Juárez-Vicuña Y., Sánchez F., Martínez-Martínez E. (2016). Inflammation Related MicroRNAs Are Modulated in Total Plasma and in Extracellular Vesicles from Rats with Chronic Ingestion of Sucrose. BioMed Res. Int..

[136] Guan X., Gao Y., Zhou J., Wang J., Zheng F., Guo F., Guang A., Li X., Wang B. (2015). miR-223 Regulates Adipogenic and Osteogenic Differentiation of Mesenchymal Stem Cells Through a C/EBPs/miR-223/FGFR2 Regulatory Feedback Loop. Stem Cells.

[137] Jeong Kim Y., Jin Hwang S., Chan Bae Y., Sup Jung J. (2009). MiR-21 regulates adipogenic differentiation through the modulation of TGF-?? signaling in mesenchymal stem cells derived from human adipose tissue. Stem Cells.

[138] Kang M., Yan L.-M., Zhang W.-Y., Li Y.-M., Tang A.-Z., Ou H.-S. (2013). Role of microRNA-21 in regulating 3T3-L1 adipocyte differentiation and adiponectin expression. Mol. Biol. Rep..

